# A Rare Case of Primary Mucinous Cystadenoma of Spleen

**DOI:** 10.4021/jocmr2009.09.1257

**Published:** 2009-10-16

**Authors:** Onkar Singh, Shilpi Gupta, Sumit Shukla, Raj Kumar Mathur

**Affiliations:** a Department of surgery, MGM Medical College & MY Hospital Indore, 452001, India.

## Abstract

Mucinous cystadenomas are relatively uncommon benign cystic tumors. Most of them are found in the ovary, pancreas, and appendix. Primary mucinous cystadenoma of spleen is extremely rare and only countable cases have been recorded. These are assumed to arise from heterotopic pancreatic tissue or invaginated spleenic capsular mesothelium. We present a rare case of primary mucinous cystadenoma of spleen without pancreatic tissue in it. Exact tissue of origin could not be determined.

**Keywords:**

Cystadenoma; Splenic cyst; Pseudomyxoma peritonei

## Introduction

Mucinous cystadenomas are relatively uncommon benign cystic tumors. Most of them are found in the ovary, pancreas, and appendix. However, they have also been identified in other unusual sites such as retroperitoneum, fallopian tube, lung, urinary bladder, and liver. Primary mucinous cystadenoma of spleen has been reported very rarely. In these cases it was presumed that the tumor arose either from invaginated capsular mesothelium of the spleen or from heterotopic pancreatic [1,2] or enteric tissue within the spleen [3,4]. We report an extremely rare case of primary mucinous cystadenoma of spleen. Only few cases have been documented in the literature [3-7].

## Case Report

**Figure 1:**
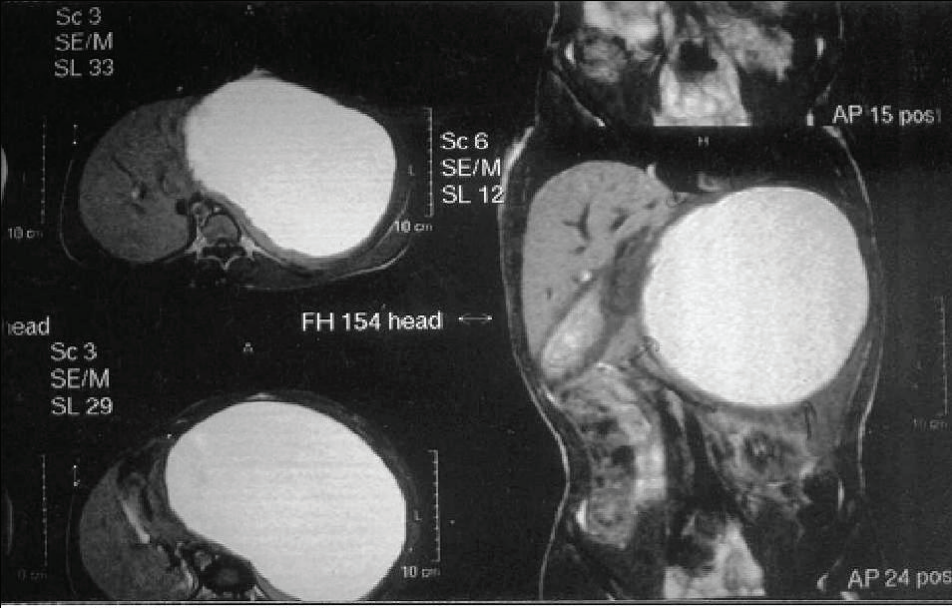
MRI demonstrating a large well-defined, unilocular, cystic, encapsulated lesion of spleen.

A 22-year-old male presented with dull aching pain associated with a slow growing lump in left hypochondrium for 3 months. Physical examination revealed a well-defined ovoid, nontender, non pulsatile, smooth surfaced mass of size approximately 24 x 16 cm, that involved the left hypochondrium, epigastrium and umbilical regions, and was moving with respiration. Ultrasonography (USG) abdomen suggested inferiorly displaced spleen due to a large well-defined hypoechoic cystic mass of size 15 x 10 cm. Barium meal examination showed extra luminal indentation of left lateral wall of stomach. Passage of barium through stomach and duodenum was smooth. Magnetic resonance imaging (MRI) demonstrated a large well-defined, unilocular, cystic, encapsulated lesion of size 20.2 x 19.3 x 15.1 cm in left subdiaphgramatic region, arising form spleen, with hyper intense signal on both T1 and T2, producing marked compression and displacement of adjacent structures (Fig. 1). Pancreas, appendix and liver were normal.

**Figure 2:**
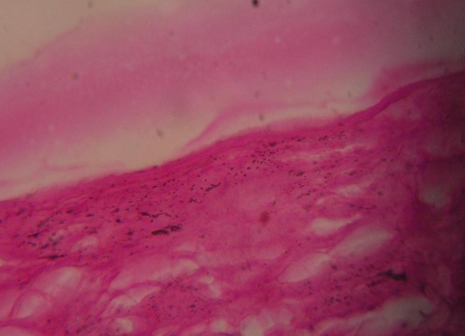
Histopathological examination showing unilocular mucinous cystadenoma of spleen.

On exploratory laparotomy, a tense and cystic mass, of size 2015 cm, arising from the diaphragmatic surface of spleen, involving the whole left hypochondrium with extension into the umbilical region and epigastric region displacing the stomach to right side, was found. Other common sites for cystic lesion like pancreas, appendix, mesentery, and liver were found normal. Splenectomy was done. The cut surface showed unilocular cystic mass with smooth inner surface and profuse insipissated mucus. Microscopically, papillary structure was found on the inner surface of cystic space, which was lined by a single layer of mucin-producing epithelial cells without malignant change. The splenic tissue was outside the cystic wall. Histopathological examination was suggestive of unilocular mucinous cystadenoma of spleen (Fig. 2). No pancreatic tissue was found in the specimen. Patient was discharged one week later. Abdominal USG demonstrated no recurrence one year later.

## Discussion

Splenic tumors are uncommon neoplasms. Morgenstern et al. classified splenic tumors into four categories: lymphoid tumors, non-lymphoid tumors, metastatic tumors, and tumor-like lesions, such as cysts and hamartomas. The most common non-lymphoid tumors are vascular tumors. However, primary splenic tumors arising from epithelial origin are extremely rare [8].

Splenic mucinous cystic tumors are defined as cystic spaces lined by mucin-producing columnar cells and ranged from benign cystadenoma to frankly malignant cystadenocarcinoma. Primary mucinous cystadenoma of spleen is an extremely rare finding [3,4,6,7]. The exact histogenetic mechanism of splenic mucinous cystic tumors is unknown except those arising from heterotopic pancreatic tissues [1]. However, mucinous cysts are usually found in association with the cystadenoma of pancreas, pseudomyxoma peritonei and mucocele of appendix [7].Only few cases, in which the tissue of origin was un-defined, are documented in the literature [3-7].

Simple mucinous cyst of spleen is a rare and close differential diagnosis, which may rarely present as pseudomyxoma peritonei [9]. Distinction is difficult to make because of low incidence and nonspecific imaging finding. Ultrasound and MRI scan, in case of mucinous cystadenoma may demonstrate a cystic lesion which may or may not contain an internal septum. Anyway, definitive diagnosis is made by surgical exploration and histopathology. Only few cases of primary mucinous cystadenoma of spleen have been reported in which tissue of origin was undefined although pseudomyxoma peritonei may uncommonly present as splenic metastasis [10]. In our case, pancreas was normal and there were no definite heterotopic pancreatic tissues in and around the splenic lesion. So, the exact histogenetic mechanism was not known. Going by Morinagas presentation [3], we concluded that the primary splenic mucinous cystadenoma may originate in the invagination of the splenic capsular mesothelium.

In conclusion,a rare case of primary mucinous cystadenoma of spleen is presented. Tissue of origin may or may not be identifiable, but it is hetertopic pancreas usually or invaginated splenic capsular mesothelium.
